# Milling of Graphene Reinforced Ti6Al4V Nanocomposites: An Artificial Intelligence Based Industry 4.0 Approach

**DOI:** 10.3390/ma13245707

**Published:** 2020-12-14

**Authors:** Mustafa M. Nasr, Saqib Anwar, Ali M. Al-Samhan, Mageed Ghaleb, Abdulmajeed Dabwan

**Affiliations:** 1 Industrial Engineering Department, College of Engineering, King Saud University, Riyadh 11421, Saudi Arabia; sanwar@ksu.edu.sa (S.A.); asamhan@ksu.edu.sa (A.M.A.-S.); mageed.ghaleb@ryerson.ca (M.G.); adabwan@ksu.edu.sa (A.D.); 2 Department of Mechanical and Industrial Engineering, Ryerson University, Toronto, ON M5B 2K3, Canada

**Keywords:** Ti6Al4V matrix nanocomposite, graphene nanoplatelets, multi-objective particle swarm optimization, artificial intelligence, industry 4.0

## Abstract

The studies about the effect of the graphene reinforcement ratio and machining parameters to improve the machining performance of Ti6Al4V alloy are still rare and incomplete to meet the Industry 4.0 manufacturing criteria. In this study, a hybrid adaptive neuro-fuzzy inference system (ANFIS) with a multi-objective particle swarm optimization method is developed to obtain the optimal combination of milling parameters and reinforcement ratio that lead to minimize the feed force, depth force, and surface roughness. For achieving this, Ti6Al4V matrix nanocomposites reinforced with 0 wt.%, 0.6 wt.%, and 1.2 wt.% graphene nanoplatelets (GNPs) are produced. Afterward, a full factorial approach was used to design experiments to investigate the effect of cutting speed, feed rate, and graphene nanoplatelets ratio on machining behaviour. After that, artificial intelligence based on ANFIS is used to develop prediction models as the fitness function of the multi-objective particle swarm optimization method. The experimental results showed that the developed models can obtain an accurate estimation of depth force, feed force, and surface roughness with a mean absolute percentage error of 3.87%, 8.56%, and 2.21%, respectively, as compared with experimentally measured outputs. In addition, the developed artificial intelligence models showed 361.24%, 35.05%, and 276.47% less errors for depth force, feed force, and surface roughness, respectively, as compared with the traditional mathematical models. The multi-objective optimization results from the new approach indicated that a cutting speed of 62 m/min, feed rate of 139 mm/min, and GNPs reinforcement ratio of 1.145 wt.% lead to the improved machining characteristics of GNPs reinforced Ti6Al4V matrix nanocomposites. Henceforth, the hybrid method as a novel artificial intelligent method can be used for optimizing the machining processes with complex relationships between the output responses.

## 1. Introduction

In recent years, there is an increasing demand for using the nanoparticle reinforced metal matrix nanocomposites (MMCs) in the aircraft and biomedical sectors [1]. This is due to their excellent properties such as high corrosion resistance, high specific strength, and high elastic modulus over pure metals [1,2,3]. In general, MMCs have lightweight metals such as titanium, titanium alloy, aluminum, and the reinforcements are nanomaterials in the form of particle/fiber having higher-strength. Since graphene was discovered, it has been widely used to form metals and ceramics-based nanocomposites. Graphene-based nanocomposites have been commonly reported to have higher strength, higher toughness, high hardness, excellent tribology, and improved thermal and electrical conductivity [4,5]. Besides, compared with other reinforcement materials, it was considered as easier to disperse uniformly in metal/ceramic matrix composites [6].

Recently, graphene has become a more attractive nano-reinforcement material for improving the performance of metal matrix nanocomposites. Graphene-reinforced titanium nanocomposites are one of the metal matrix nanocomposites that have been considered as promising materials for high-temperature applications. Graphene-reinforced titanium nanocomposites with different graphene contents have been successfully fabricated by powder metallurgy and rapid consolidation techniques, namely spark plasma sintering (SPS), hot isostatic pressing (HIP), hot pressing (HP), and high-frequency induction heating (HFIH) techniques. Many studies have been focused on the preparation and investigation of the mechanical properties of the consolidated graphene reinforced titanium matrix nanocomposites [2,7,8,9,10,11,12,13,14]. Thereafter, the machinability analysis of these high-performance materials becomes very necessary after their fabrication and that needs more investigation.

The machinability studies of the MMCs can be divided into investigating the machining parameters, type of material, and reinforcement materials and ratio. Most of the machining related work regarding the MMCs is focused on the machining parameters of the aluminum-based composites with different reinforcement materials (SiC, TiC, MgO, B_4_C, TiB_2_, and TiAl_3_). For instances, Rai et al. [15] analyzed the machinability of pure Al, Al-TiC, and Al-TiAl_3_ composites, and Al-Si alloy. They found that the cutting force for Al-TiC composite was lower than Al-TiAl_3_ composite, Al-Si alloy, and pure Al. Furthermore, the machined surfaces of Al-TiC composites exhibited a better surface finish compared to Al-TiAl_3_ composites and Al-Si alloy. The good performance of Al-TiC composite was contributed to the presence of TiC particles, which facilitate the formation of microcracks during the formation of the chip resulting in lower cutting forces. Rajeswari and Amirthagadeswaran [16] conducted experiments to study the effects of weight percentages of SiC, feed rate, depth of cut, and spindle speed on cutting force, tool wear, and surface roughness of fabricated SiC/Al_2_O_3_/Al hybrid composites. Based on the analysis of variance (ANOVA), feed rate, depth of cut, spindle speed, and weight percentages of SiC were found to have a significant effect on the output responses. Jiang et al. [17] investigated the machinability of TiB_2_ particles reinforced aluminum metal matrix composite. The effects of spindle speed and feed rate on cutting force and surface roughness during the turning process were analyzed. They found that the machining forces for TiB_2_/Al MMCs were slightly higher and the surface roughness was lower than that for the non-reinforced alloy with increasing cutting speed mainly due to the material flow. Pramanik et al. [3] studied the machinability of SiC/Al nanocomposite with 10% volume fraction of SiC particles by face milling and investigated the effect of cutting speed and feed rate on surface roughness, machining forces, and chip surface. They found that chip ratio, surface roughness, and machining forces were affected by feed rate and cutting speed. Ekici and Gülesin [18] studied the machinability of B_4_C/Al composite during milling with 5%, 10% and 15% ratio of B_4_C particles. They found that the cutting forces during milling B_4_C/Al composite were increased with the increase in cutting speed and B_4_C reinforcement ratio when carbide cutting tools were used. In the case of the cubic boron nitride tools, the milling forces decreased when the cutting speed decreased, and the B4C reinforcement ratio increased. Pul [19] analyzed the machinability of MgO/Al composite with ratios of 5%, 10%, and 15% of MgO particles by turning. They found that the most favourable results were obtained with a MgO reinforcement ratio of 10 wt.% MgO and using carbide cutting tools.

It is worth mentioning that titanium and its alloys are considered very difficult to machine materials [20,21]. This means that the machining of titanium metal matrix composites is also very challenging. Regarding the machining of titanium metal matrix composite, limited work has been reported in the previous studies. For example, Ding et al. [22] performed the grinding of the TiCp/TiBw/Ti6Al4V composites, and pure Ti6Al4V alloy. They found that the grinding forces were much higher for the composites than the pure Ti6Al4V alloy. This was due to the differences in elasticity modulus of the machined materials. Aramesh et al. [23] investigated the tool wear mechanisms of the cubic boron nitride inserts during turning of 10–12 vol% TiC/Ti6Al4V matrix composite. Niknam et al. [24] studied the machinability of TiC/Ti6Al4V matrix composites during turning process under different depths of cut, cutting speed, and feed rate. They found that the cutting speed and feed rate are the major machining parameters affecting the surface roughness and flank wear.

Regarding the machinability analysis, very limited work has been reported on the machining analysis of the graphene-based metal matrix nanocomposites. Gao and Jia [1] developed a finite element model to study the effect of the machining parameters and reinforcement ratio, namely cutting depth, GNPs weight fraction, and GNPs average size on the average cutting force during micro-machining of GNPs/Mg nanocomposite. They found that weight fraction, and GNPs average size affect the output responses. Abdulgadir et al. [25] reported an experimental study on the drilling of the Mg-nanocomposite reinforced by 10 wt.% SiC and 0.25 wt.% GNPs. They found that the presence of the GNPs in Mg-nanocomposite reduces the friction between the cutting tool and nanocomposite due to the lubricant effect of graphene resulting in decreasing cutting force. Na et al. [26] performed the micromachining of the graphene nano-flakes (GNF) reinforced aluminum nanocomposite. The effect of feed rate and GNF contents on milled surface morphology, force component, and chip formation were investigated. The obtained results showed that all output responses were considerably affected as the graphene contents increased compared with base aluminum.

It should be noted that the selection of the optimal cutting conditions for machining metal matrix composites plays a critical role in green and sustainable manufacturing where efforts are made to minimize tools and energy consumption [27,28]. In addition, in ascertaining the quality of machined parts, minimizing machining cost and increasing productivity. Therefore, several researchers used traditional methods for optimizing the cutting conditions such as Taguchi method [29] and Response surface methodology (RSM) [30,31]. However, Taguchi and RSM methods obtain optimal solutions dependent on the randomly chosen initial solutions, and the optimization falls into the local solution [32,33]. On the other hand, metaheuristic algorithms are being proposed by researchers to guarantee a globally optimal solution for machining characteristics. For example, [17] optimized the cutting condition during turning of TiB_2_/Al for maximizing MRR and minimizing the surface roughness using a genetic algorithm (GA). Gupta et al. [34] optimized the cutting condition during the turning of Ti6Al4V for minimizing cutting forces, surface roughness, and tool wear using response surface methodology and particle swarm optimization. Choudhary et al. [35] used hybrid particle swarm optimization and genetic algorithm for optimizing submerged arc welding process parameters. According to [32] GA method has some limitations, including higher computation time, too many control parameters, and deliberate convergence. To overcome these limitations, a multi-objective particle swarm optimization (MPSO) is adapted as an effective tool to optimize cutting parameters. MPSO is faster compared to the GA method, and can simultaneously apply both global and local search, whereas GA is mainly more effective for global search, as mentioned by [32,34,36,37,38]. The effectiveness of the optimization methods depends on the prediction model as a fitness function. Extensive work has been performed on using response surface methodology and factorial design to develop mathematical models as fitness functions. However, these models may not be able to ensure reliable results because the machining processes are very complex with nonlinear behaviours. Therefore, there is an increasing interest in the development of models for machining processes to guarantee and obtain reliable results. Artificial intelligence techniques are effective tools to develop models for complex nonlinear systems [32,39]. Bustillo et al. [40] developed prediction models for flatness deviation of the milled surface during face milling of AISI 1045 steel using the machine learning method. Abbas et al. [41] developed prediction models for surface roughness, machining time, machining cost during the turning of AA6061 alloy by using a neural network.

Artificial intelligence techniques based on adaptive neuro-fuzzy inference systems (ANFIS) provides more realistic results as compared to neural network and mathematical models based on the design of experiments [42,43,44,45,46]. Moreover, employing the integrating artificial intelligence methods as fitness function during optimization presented accurate results compared with conventional method (Taguchi and RSM methods). Conde et al. [39] developed prediction models using an artificial neural network and used these models as a fitness function for simulated annealing for optimizing the wire electrical discharge machining. Gopan et al. [32] combined an artificial neural network with the particle swarm optimization (PSO) method for optimizing the cutting parameters to minimize the surface roughness and cutting forces during the grinding process and found accurate results during validation. Abbas et al. [47] optimized the cutting condition during the face milling of high-strength steel grade-H for minimizing surface roughness and machining time using an artificial neural network with the Edgeworth-Pareto method.

On the other hand, many industries are using artificial intelligence techniques to enable smart automated manufacturing. Complex nonlinear machining processes can greatly benefit if they shift towards smart machining. This will require adjusting the machining parameters autonomously and adapting to current conditions to achieve higher performance. Therefore, to satisfy the requirements of smart machining in Industry 4.0, artificial intelligence and machine learning-based prediction models of machining processes are a fundamental requirement.

It is evident from the literature review that the graphene nano-reinforcement materials enhance the functionality and machinability of metal matrix nanocomposites. It was also found that no study has been conducted to optimize the cutting parameters by integrated ANFIS with a multi-objective particle swarm optimization (MOPSO) method for any machining process to fulfill the industry 4.0 smart manufacturing requirements [48,49]. It should be noted that no previous work has been reported on optimizing the machining parameters for machining GNPs-Ti64 nanocomposites. Only recently a study has been reported [50], where the machinability of the GNPs reinforced Ti6Al4V nanocomposites was presented. This study explored the effect of the GNPs and milling parameters on the machining performance. They found that despite improved mechanical properties (high hardness), GNPs reinforced Ti6Al4V nanocomposites showed better machining performance. However, their work did not consider the optimization of the GNPs reinforcement ratio and milling parameters for better machining performance. The objective of this study is to develop a novel method based on ANFIS and MOPSO to optimize milling parameters and GNPs reinforcement ratio for machining GNPs-Ti64 nanocomposites. To achieve this objective, firstly, high-density hybrid GNPs-Ti64 nanocomposites are fabricated with different graphene contents by using the high-frequency induction heating (HFIH) technique. Secondly, the milling experiments are conducted based on full factorial design to study the effect of cutting speed, feed rate, and GNP reinforcement ratio on the depth force, feed force, and surface roughness. Thirdly, ANFIS models are developed based on the training and testing data, and then compared with the quadratic model to ensure the realistic results of the developed ANFIS model. Finally, multi-objective optimization was performed using hybrid ANFIS with the MOPSO method to select the optimal machining parameters and GNPs reinforcement ratio for enhancing the machining of the hybrid GNPs-Ti64 nanocomposites.

## 2. Experimental Methods

### 2.1. Production

For developing the GNPs-Ti64 nanocomposites, commercial Ti6Al4V (Ti64) alloy powder (~71 µm, ARCAM AB, Mölndal, Sweden) was used as the matrix material. The chemical composition of the received Ti64 powder is given in Table 1. Graphene nanoplatelets (GNPs) from XG Sciences, Inc., Lansing, MI, USA were used as the reinforcement material. The characteristics of GNPs are listed in Table 2.

**Figure 1:**
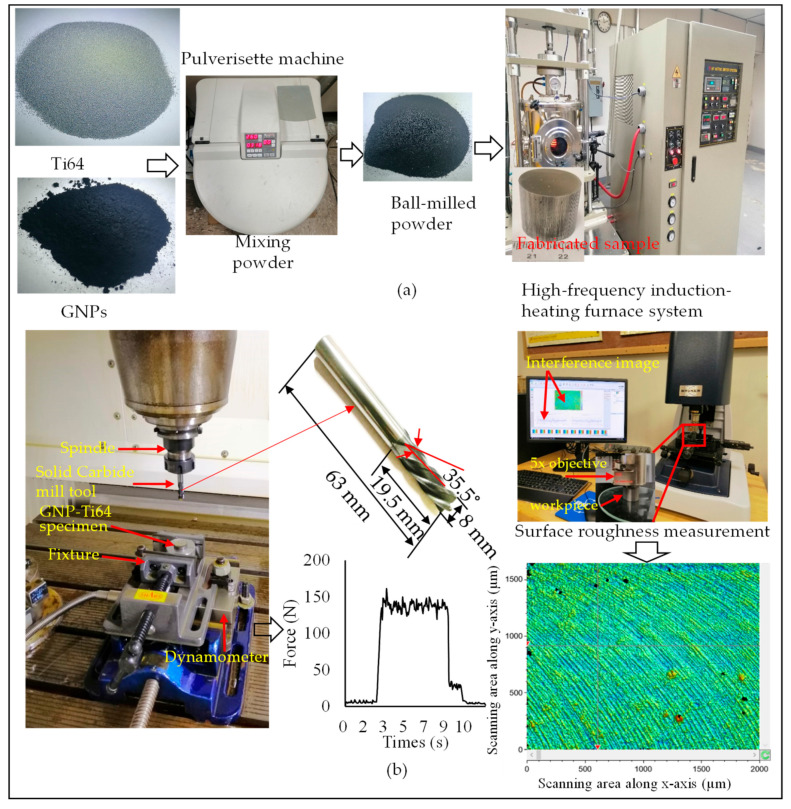
Experimental set-up and measuring devices (**a**) Preparation process for rapid fabrication of GNPs-Ti64 nanocomposite specimens (**b**) milling and measurements setups.

GNPs-Ti64 nanocomposites were fabricated by using different weight percentages of reinforcement such as 0 wt.%, 0.6 wt.%, and 1.2 wt.% GNPs. All GNPs powders were mixed into Ti64 through a Pulverisette ball mill machine (FRITSCH GmbH, Idar-Oberstein, Germany). The milling process was performed at 100 rpm for 4 h under a ball-to-powder weight of 10 [51]. The obtained ball-milled powder was loaded into a graphite die with an internal diameter of 20 mm and then consolidated by HFIH furnace (HFActive Sinter System, ELTEK, Dongan-gu, South Korea) The consolidation was performed at a temperature of 1000 °C, a heating rate of 200 °C/min, and a uniaxial pressure of 50 MPa [52]. Process flow diagram of the fabrication process of GNPs-Ti64 nanocomposites is illustrated in Figure 1a. After fabrication, all specimens were ground using SiC papers with P200, P200, P400, P600, P800, P1000, P1500, and P2000 grit size, followed by polishing with Al_2_O_3_ suspension, and etched for 10 s by Kroll’s solution. Then, a scanning electron microscope (SEM) from Jeol, Tokyo, Japan (Model JCM 6000Plus) was used to examine the dispersion of GNPs in the Ti64 matrix.

### 2.2. Machining Setup and Measurements

**Figure 2:**
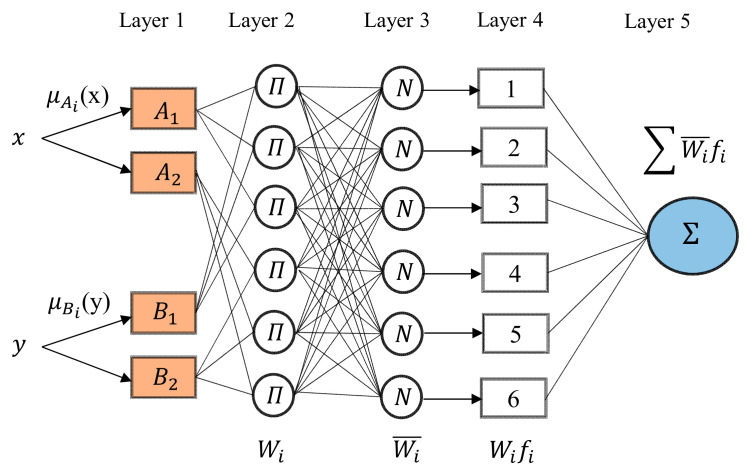
Architecture of ANFIS model [54].

Machining tests of the GNPs-Ti64 nanocomposites were carried out after the fabrications were completed successfully. Milling experiments were performed on the DMC 635 V three axes CNC vertical milling machine (DMG Mori, Oelde, Germany) with the maximum tool rotational speed of 8000 rpm and feed rate of 24 m/min. The experimental setup consists of a CNC vertical milling machine equipped with a dynamometer, as shown in Figure 1b. Kistler dynamometer (Kistler Corp, Winterthur, Switzerland) type 9257B with charge amplifier (type 5070A), acquisition system (type 5697A1), and DynoWare’ software type 2825D-02 (DynoWare, version 2.4.3.2, Kistler Corp, Winterthur, Switzerland) were used to measure the forces. Solid carbide end-mill cutting tools with 8 mm diameter, four flutes (helix angle 35°), axial rake angle 5.5°, and radial rake angle of 9° was used for milling, as shown in Figure 2. Each experiment was repeated two times and the average of the measured forces during two experiments was used later. A 3D optical profilometer (Contour GT-K, Bruker, Berlin, Germany) was used to measure the roughness of the milled surfaces. The roughness of the machined surface was measured in terms of surface area (Sa). The roughness measurement was repeated seven times for each milling experiment, and later the average value was used. Durascan 10HV (Struers A/S, Ballerup, Austria) was used to measure the Vickers hardness of the fabricated samples by using a load of 0.5 Kg for a dwell time of 10 s. For each sample, the hardness measurement was repeated five times and later the average value was used.

### 2.3. Experimental Design

A full factorial design was applied to identify the experimental conditions and investigate the effect of machining parameters on the output responses. Three cutting parameters, namely cutting speed (V), feed rate (f), and reinforcement ratio (R) were selected as the machining factors. Depth force (F_d_), feed force (F_f_), and surface roughness (SR) were used as output responses to evaluate the machining characteristics of the fabricated GNPs-Ti64 nanocomposites. The purpose of this study is to reduce the cutting force components and roughness of the milled surface. The range of the selected parameters for the milling GNPs-Ti64 nanocomposites was selected from the previous study [50,53]. The selected cutting parameters and their ranges are presented in Table 3.

Statistical analysis based on analysis of variance (ANOVA) was used to estimate the effects of selected parameters and their interaction on the output responses. The statistical analysis software Minitab 17 was used to systematically analyze the effects of cutting speed, feed rate, and graphene content on the cutting force components, and surface roughness. The coefficient of determination (R^2^) and correlation coefficient (R) were used to evaluate the accuracy of the models.

### 2.4. Hybrid ANFIS-MOPSO Approach

#### 2.4.1. Adaptive Neuro-fuzzy Inference System Model

ANFIS is an effective method to develop prediction models for solving very complex and nonlinear processes. ANFIS combines fuzzy inference system (FIS) and artificial neural network (ANN). FIS consists of five networks and each layer is described by several node functions. Figure 2 shows the ANFIS model structure with two membership functions (MFs) for each input and one output, as explained in ref [54]. The ANFIS was implemented via MATLAB 2016a (MATLAB, 16b, MathWorks Inc., Natick, MA, USA).

The meaning of each layer in fuzzy inference system is described as follows [54];

Layer 1: Fuzzification layer, in this layer a membership value is computed by using the following equation.

(1) $$\mu Ai\left(x\right)=\frac{1}{1+{\left[{\left(\frac{x-c_{i}}{a_{i}}\right)}^{2}\right]}^{b_{i}}}$$

where $\;\mu \;Ai\left(x\right)$ represents the membership function, a_i_, b_i_, and c_i_ form a parameter set that changes the forms of the fuzzy membership with a value between 0 and 1.

Layer 2: Product layer, in this layer the incoming signals are multiplied and sent out

*i* = *µAi(x) × µBi(y)*, *i* = 1, 2 (2)

where $\;\mu \;Bi\left(x\right)$ represent the membership function

Layer 3: Normalizing layer, in this layer, the normalized firing strength is calculated using the following equation:

(3) $${\bar{w}}_{i}=\frac{w_{i}}{\sum_{i}w_{i}},\;i=1,2$$

where, $w_{i}$ denotes the output of each layer.

Layer 4: This layer is called defuzzification layer where every ith node in this layer is expressed with the following equation:

(4) $${\bar{w}}_{i}.f_{i}={\bar{w}}_{i}.\left(p_{i}.x+q_{i}.y+r_{i}.z+s_{i}\right)$$

where ${\bar{w}}_{i}$ is the normalized firing strength and p_i_, q_i_, and r_i_ are the consequent parameters, which are determined using a training algorithm.

Layer 5: this is output layer and the system output is computed as follows.

(5) $$\mathrm{Output}\;=\;\sum_{i}{\bar{w}}_{i}.f_{i}=\frac{\sum_{i}w_{i}.f_{i}}{\sum_{i}w_{i}}\;,\;i=1,2$$

In this work, ANFIS prediction models were used to establish a non-linear relationship between the inputs (cutting speed, feed rate, and reinforcement ratio) and the output responses (cutting force components and surface roughness). These developed models were used to estimate the machining performance, and also as the fitness function for MOPSO to carry out the optimization procedure

#### 2.4.2. Multi-Objective Particle Swarm Optimization

Coello and Lechuga [55] extended the metaheuristic developed by [56] called “particle swarm optimization” (PSO), to optimize multi-objective problems. The extended algorithm is a multi-objective version of PSO, which is called the Multi-Objective Particle Swarm Optimization (MOPSO). The proposed MOPSO uses the Pareto Dominance principle to evaluate the particle travel trajectory and preserves previously defined non-dominated vectors in a global repository that are later used by other particles to control their own path [55]. To handle the optimization process of the multi-objective problems, MOPSO incorporates a global repository and a geographically based system, which is inspired by the external file used with the Pareto Archived Evolution Strategy (PAES).

Similar to PSO, particles in MOPSO exchange knowledge and continuously shift towards both memories, the global best particles and their local best. Nevertheless, unlike PSO, there is more than one objective to optimize (i.e., find and determine the global or local best). The non-dominated particles in the swarm are collected into a sub-swarm called Repository, and each particle selects its global best target among the members of this Repository. Domination and probabilistic based rules are utilized to determine the particle’s local (or personal) best.

**Figure 3:**
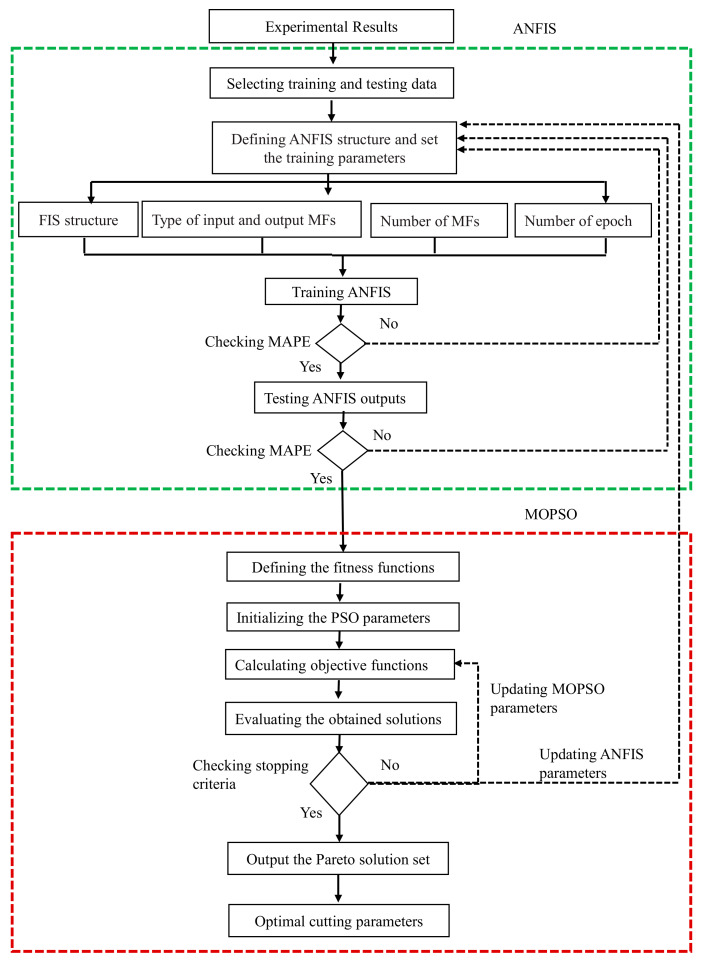
Structure of a hybrid novel ANFIS-MPOSO approach.

As the effectiveness of the MOPSO in achieving reliable results is associated with the proper formulation of fitness functions [55]. Thus, the fitness function should precisely map the input values and output responses. Considering that conventional mathematical models often tend to fail when mapping certain complex processes, an ANFIS based on artificial intelligence will be the best alternative as a fitness function for MOPSO algorithm. The overall structure of the hybrid method is illustrated in Figure 3. Details of the hybrid approach shown at the following steps:

Step 1: Identify the prediction models for F_d_, F_f_, and SR responses which are used as fitness functions of MOPSO. In ANFIS training, experimental results and input information of ANFIS are used to identify the training parameters that achieve high accuracy predictive models for selected outputs responses. The training parameter will be updated until obtaining the minimum MAPE. More details of the input learning information are provided in Section 3.3.

Step 2: In ANFIS testing, experimental results were used to evaluating the ANFIS based on MAPE. The MAPE will be checked for all testing data to achieve minimum errors or updated the ANFIS training to obtain the best setting parameters for ANFIS learning.

Step 3: Define the fitness functions and the constraints for F_d_, F_f_, and SR responses according to the developed ANFIS model in the previous steps. First, the initializing MOPSO parameters are setup to execute the optimization steps. Then, the objective functions were evaluated using Pareto solution set and MOPSO parameters were updated until obtaining good convergence characteristics of MOPSO. In addition, the ANFIS parameters are updated to get better convergence of MOPSO results.

Step 4: Evaluating the obtained Pareto solution set to select the optimum parameters set which satisfy the purpose of this study.

### 2.5. Desirability Approach

The desirability approach is a statistical technique for solving multi-objective problems. It evaluates how well a combination of input parameters meets the desired goal set for the output responses. This is done by converting each predicted output response into a desirability number varying from 0 to 1. The desirability value approaching 0 shows that the goal has not been achieved whereas the value approaching 1 indicates that the best outcome has been achieved for the given combination of the input parameters. It should be noted that when more than one output response is optimized together for a single set of input parameters the calculated overall desirability is called composite desirability (D). A commercially available software (Minitab) offers the desirability approach for optimizing the input parameters. The results of the desirability approach were compared with the newly developed Hybrid ANFIS-MOPSO approach.

## 3. Results and Discussions

### 3.1. Microstructure and Hardness of the Produced Nanocomposites

**Figure 4:**
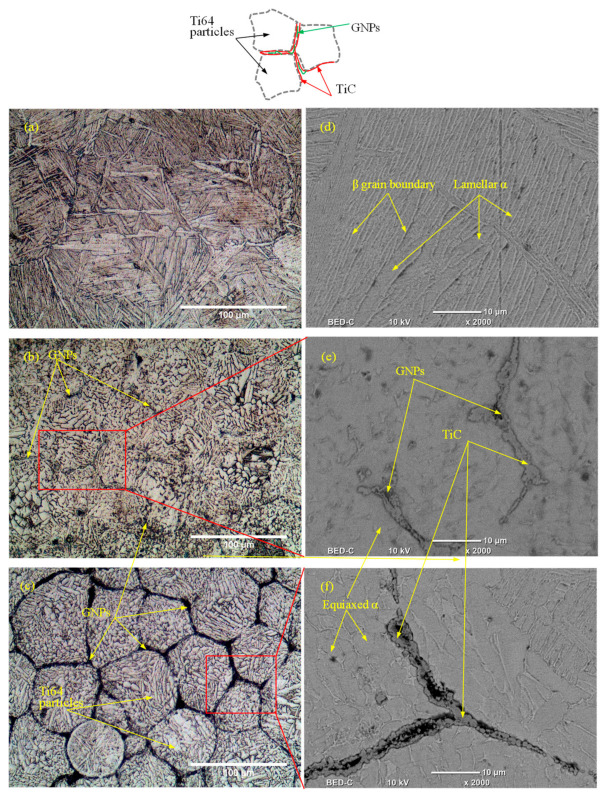
Microstructure images of the fabricated samples (**a**,**d**) Pure-Ti64; (**b**,**e**) 0.6 wt.% GNPs, (**c**,**f**) 1.2 wt.% GNPs.

Figure 4a–c shows optical microscopy and Figure 4d,e shows SEM backscattered images of the produced GNPs-Ti64 nanocomposites with different graphene reinforcement ratio. It can be seen from Figure 4a that the microstructure of pure Ti6Al4V is characterized by α + β lamellar structure. For the fabrication of the GNPs-Ti64 nano-composites, it was necessary that the GNPs reinforcement must be uniformly dispersed in the Ti64 matrix. Figure 4b,c show the GNPs reinforcements are uniformly distributed between the Ti6Al4V matrix particles. In addition, the nanocomposite with 0.6 wt.% GNPs were characterized by coarse equiaxed α structure with refined lamellar α + β structure. Similarly, the nano-composite with 1.2 wt.% GNPs was characterized by equiaxed α. Nasr et al. [50] presented in details how these differences in microstructure were developed after adding the GNPs reinforcements to Ti64. Moreover, the SEM backscattered images on the selected area were taken to show the formed TiC and graphene agglomerates during sintering at high temperature, as shown in Figure 4d–f. More details on the effect of GNPs reinforcement on microstructure were discussed in the previous study [50].

The hardness of the fabricated specimens is shown in Table 4.

It can be concluded that the highest hardness was obtained at 0.6 wt.% GNPs reinforcements due to the developed hard phases (coarse equiaxed microstructure and TiC hard particles). Afterwards, with increasing the GNPs reinforcements to 1.2 wt.% GNPs, the hardness was decreased due to the agglomeration of the graphene around the Ti64 matrix particles.

### 3.2. Statistical Analysis

Table 5 presents the experimental results of the machining GNPs-Ti64 nanocomposites specimens under milling parameters and GNPs contents. In order to analyze the results of the experiments to estimate the effect of machining parameters and GNPs reinforcement ratio on all the output responses, an ANOVA was performed with a confidence interval of 95%. *p*-value was used to check the statistical significance of the parameters. The terms with *p*-value less than 0.05 have a significant effect on the outputs [57]. Furthermore, mathematical models based on the response surface were developed to establish the relationship between inputs and outputs, which were used for evaluating the effectiveness of ANFIS models.

Table 6 presents the results of the ANOVA for depth force, feed force, and surface roughness. For the depth force, it can be found that cutting speed (V), feed rate (F), and the second-order term of reinforcement ratio (R), cutting speed (V), and the interactions of V and F were significant terms. Regarding, the feed force (F_f_), the results indicated that the terms of the cutting speed (V), feed rate (F), and the second-order term of reinforcement ratio (R), and feed rate (f) were significant terms, as shown in Table 6. In addition, the interaction between V and f has a significant effect on feed force. Similarly, the ANOVA results for the surface roughness indicated that speed (V) and feed rate (F), GNPs reinforcement ratio (R), and the interactions between V and R were significant terms, as shown in Table 6. The second-order term of reinforcement F has a significant effect on surface roughness.

The coefficient of determination (R^2^) for each RSM model was calculated as 86.40%, 88.27%, and 90.73% for depth force, feed force, and surface roughness models, respectively. It can be inferred that the model has a high significance of 86.40%, 88.27%, and 90.73%.

**Figure 5:**
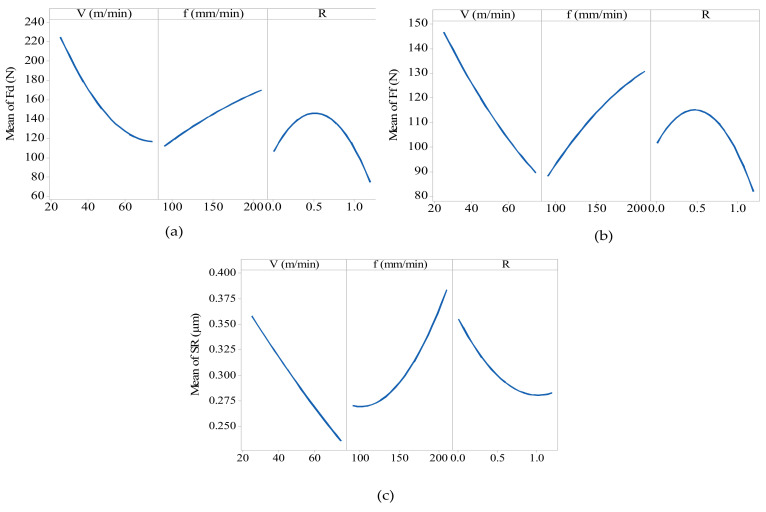
Effects of the milling parameters and GNPs reinforcement ratio on the (**a**) depth force (**b**) feed force and (**c**) surface roughness.

In addition, the main effect plots were obtained for each output response, as shown in Figure 5. Figure 5a,b shows that the depth force and feed force was affected by cutting parameters and GNPs reinforcement ratio. With the increasing cutting speed, the depth and feed forces decreased. This is attributed to thermal softening due to an increase in the cutting speed [58]. With an increase in the feed rate, the depth and feed force increased. This is attributed to an increase in friction between the cutting tool and workpiece [58,59]. Similarly, an increase in the reinforcement ratio to 0.6 wt.%, the feed and depth force increased due to the presence of the equiaxed grains in the Ti64 matrix, and developed TiC hard particles. When the GNPs reinforcement ratio increased to 1.2 wt.%, the feed and depth force decreases. This happens because of the lubricant effect of graphene which led to a reduction in the fraction between tool and workpiece. In the case of the surface roughness, as shown in Figure 5c, the roughness of the milled surface increased with the increasing cutting speed and feed rate, as previously reported [58,59]. In addition, the roughness of GNPs-Ti64 nanocomposite are affected by the GNPs reinforcement ratio, and decreases with increasing the GNPs ratio from 0.6 wt.% to 1.2 wt.%. This happened because of the development of the GNPs agglomerates and TiC particles surrounding the Ti64 matrix particles, both of which ease the propagation of micro-cracks [15]. In addition, the GNPs-Ti64 nanocomposites with 0.6 wt.% GNPs presented higher cutting force because of higher hardness compared with other samples. The roughness decreases with the rise in the GNPs wt.% because the proportion of both GNPs agglomerates and TiC particles increases with the increase in the GNPs wt.%.

The mathematical model has been developed based on the design of experiments for depth force, feed force, and surface roughness. The fit mathematical equations for all the selected responses (F_d_, F_f_, and SR) are presented in the following equations:

F_d_ = 143.7 − 4.46 V + 1.142 f + 149.6 R + 0.0435 V × V − 142.6 R × R − 0.01301 V × F (6)

F_f_ = −3.4 − 0.015 V + 1.496 F + 53.8 R − 0.00260 F × F − 55.1 R × R − 0.00722 V × f (7)

SR = 0.4827 − 0.002443 V − 0.00143 F − 0.0339 R + 0.000009 F × F + 0.0720 R × R − 0.000751 F × R(8)

### 3.3. Development of Predictive Model using ANFIS

The ANFIS models were developed for the selected milling responses (F_d_, F_f_, and SR) for GNPs-Ti64 nanocomposites. The training data was used for establishing the ANFIS models, and the testing data was used for measuring the effectiveness of the developed models. Experimental results were divided as the data was used for the training and the remaining data for the testing. To design the ANFIS model, the initial parameters were chosen for training ANFIS algorithm, as shown in Table 7.

After the training algorithm was completed, the testing data was used to validate the performance of the predictive ANFIS models. The mean absolute percentage error (MAPE) was used to validate the ANFIS performance and is obtained using the equations:

(9) $$\mathrm{MAPE}\;=\;\frac{1}{n}\sum_{t=1}^{n}|\left({\mathrm{Exp}}_{t}-{\mathrm{Pr}}_{t}\right)/{\mathrm{Exp}}_{t}|$$

During the training ANFIS algorithm, different fuzzy inference parameters were repeated until the MAPE was minimized. Table 8 presents the selected fuzzy inference parameters which achieved the lowest MAPE.

**Figure 6:**
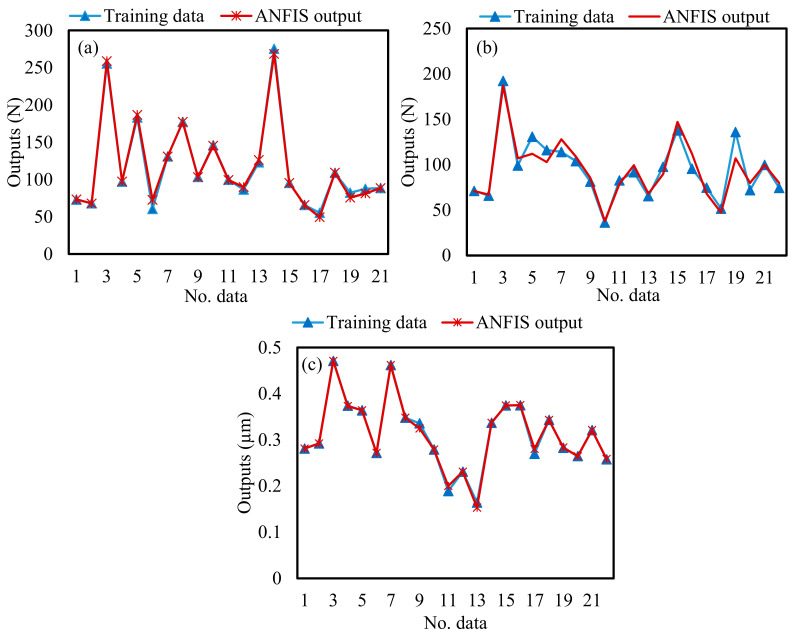
Comparison between the training data and the ANFIS outputs (**a**) depth force; (**b**) feed force; (**c**) surface roughness.

**Figure 7:**
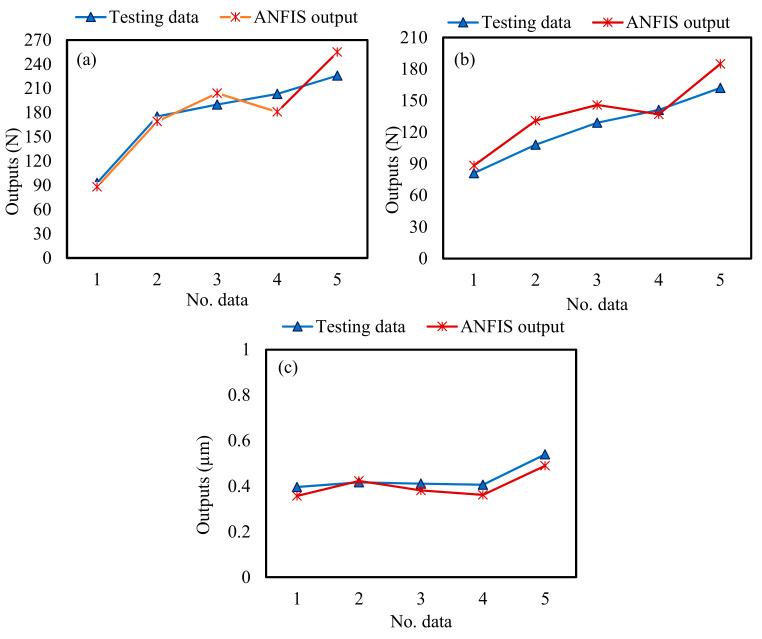
Comparison between the testing data and ANFIS outputs: (**a**) depth force; (**b**) feed force; (**c**) surface roughness.

Therefore, the training processes were applied based on the selected training parameters, as shown in Table 8. Experimental testing data was used to validate the correctness of the developed ANFIS models. Figure 6 and Figure 7 show a comparison between the experimental results and ANFIS outputs for F_d_, F_f_, and SR for the training and testing data, respectively. It can be observed that the measured and predicted values by ANFIS models are very close to each other, which indicates the ANFIS models have good robustness and can obtain an accurate fitness function for MOPSO. Moreover, it implies the correctness of the developed ANFIS models.

### 3.4. Comparison of the ANFIS Models with Mathematical Models

To evaluate the potential of the predictive ANFIS models relative to that of the quadratic models, Table 9 presents a comparison of the performance between the developed ANFIS model and quadratic model based on MAPE. It should be noted that the MAPE shown in Table 9 is the average for all the 27 experiments.

**Figure 8:**
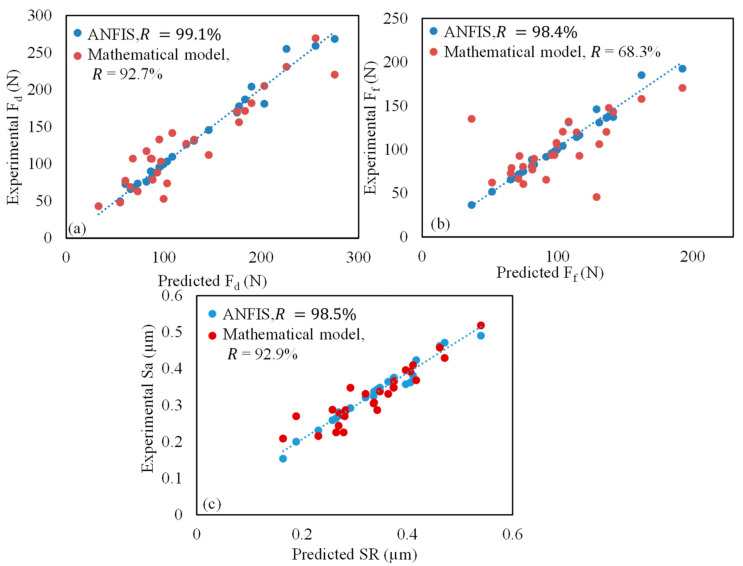
Predicted value of experimental vs. ANFIS vs. mathematical models on output responses; (**a**) depth force; (**b**) feed force; (**c**) surface roughness.

In addition, the predicted outputs values using ANFIS models are compared with the mathematical model developed by Minitab software. The comparison is represented by the residual plot in Figure 8. The efficiency is assessed by using the correlation coefficient (R) value of each model. It can be concluded that the ANFIS models for surface roughness and cutting forces presented a higher R-value compared with the mathematical model which means a good fit for predictive machining behavior.

Based on the comparison between ANFIS, and mathematical model, it can be concluded that ANFIS models performed better for all responses. Therefore, it can be concluded that the established models by ANFIS can be used efficaciously to predict the cutting force component and surface roughness during milling GNPs-Ti64 nanocomposites.

### 3.5. Multi-Objective Optimization with Hybrid ANFIS-MOPSO Approach

Minimizing the surface roughness, depth force and feed force can produce lower tool wear and better surface quality during milling GNPs-Ti64 nanocomposites. Therefore, there is a need for a single set of milling parameters (V, F) and GNPs reinforcement ratio (R) as an optimal solution for all the output responses collectively. To achieve this, multi-response optimization is required. The fitness functions for the output responses established through ANFIS technique were then employed as the objective functions for MOPSO method to find the optimal V, F, and R. Table 10 presents the MOPSO parameters and machining constrains for executing the optimization.

**Figure 9:**
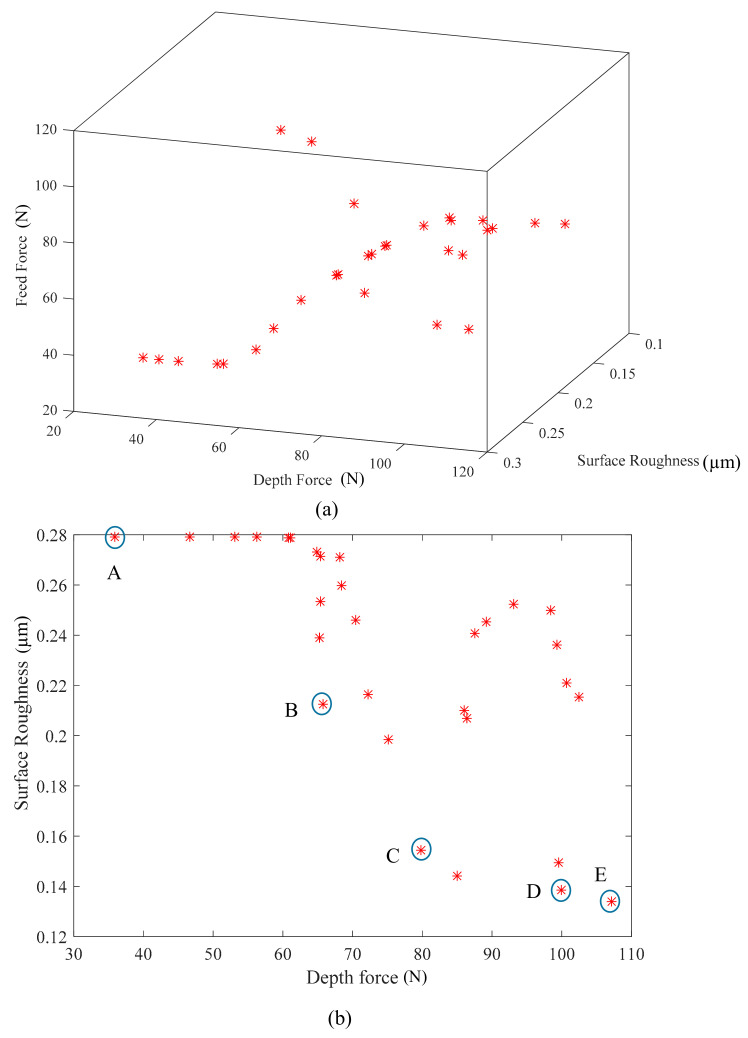
Optimal solution (**a**) 3D Pareto front solution set for objective functions (F_d_, F_f_, and SR); (**b**) 2D Pareto front graph for objective functions (F_d_ and SR).

After executing the running hybrid optimization algorithm, Pareto optimal front is plotted in Figure 9. Figure 9a shows the potential solutions which could simultaneously minimize all the output responses. In Figure 9b, five representative selected solutions (A–E) for milling parameters and GNPs reinforcement ratio of GNPs-Ti64 nanocomposites. The best solutions are plotted with a blue circle and the non-dominated ones are marked with a star circle.

Solutions at point A to E are the optimal solutions in terms of depth force, feed force, and surface roughness, respectively, as presented in Table 11. Solutions at points A to E are different trade-offs between the values of the objective. The solution at point A is the best values of depth force and feed force and the worst for the surface roughness. The solution at point B, causes an increase in the feed force and depth force as compared to the solution at point A, while this solution leads to a reduction in the surface roughness. Looking at solutions at points D and E, it can be noted that there is a large increase in the value of depth force, while there is no change in the solution values of feed force. Regarding the surface roughness at the same points (D and E), the solution values of surface roughness is slightly improved. Therefore, the obtained solutions obtained at points B and C appear to be a better compromise between depth force, feed force, and surface roughness.

These results are because of the following reasons; (i) the cutting force fluctuate due to two phases (GNPs and TiC particles) present in the GNPs-Ti64 nanocomposites, (ii) the presence of the GNPs agglomerates in GNPs-Ti64 nanocomposites led to reduce the friction between tool and workpiece due to the lubricity of the graphene (see solution point C–E in Table 10). In contrast, at low graphene reinforcement ratio, the effective lubrication becomes weak due to the scarcity of graphene sheets compared with high graphene contents (see solution points C–E in Table 11). (iii) Regarding the surface roughness, there was slight increase in the roughness at high graphene reinforcement ratio. This is because at a higher percentage of the GNPs, a thick layer of TiC particles is produced surrounding the Ti64 matrix particles. This results in encapsulation of the heat in the Ti64 particles during machining, which leads to thermal softening of the matrix material and a rise in the surface roughness. Moreover, it can be concluded that the hardness affected the machining of the nanocomposites. The nanocomposite with reinforcement ratio in range 0.6 wt.% GNPs presented higher cutting forces in all cases due to higher hardness.

### 3.6. Comparison with Commercially Available Desirability Approach Optimization

By using the developed RSM models of the depth force, feed force, and surface roughness, multi-objective optimization of milling parameters and GNPs reinforcement ratio is performed within the current range of the experimental parameters. The optimization is performed by employing the desirability function-based approach via commercially available Minitab software with aim/goal of achieving the minimum value for each output response, and to show its comparison with the newly developed hybrid ANFIS-MOPSO approach.

The optimum values of machining parameters and reinforcement ratio leading to minimum depth force, feed force, and surface roughness by using the desirability approach are listed in Table 12.

It can be found that the composite desirability of 0.9281 is achieved for multi-objective optimization, which indicates that the best outcome (i.e., minimum F_d_, F_f_, and SR) are achieved for the given combination of the machining parameters and graphene reinforcement ratio.

To further verify the reliability and accuracy of the developed ANFIS-MOPSO and desirability approach for depth force, feed force, and surface roughness, additional experiments were performed at the optimized conditions. For the ANFIS-MOPSO approach, the predicted results from the combination D were selected as it provides a combination of almost lowest roughness and intermediate cutting forces. From the comparison results in Table 13, it can be concluded that the results obtained by the hybrid ANFIS-MOPSO approach always show lower MAPE, which indicates the superior performance of this new approach as compared to the commercially available optimization desirability approach.

## 4. Conclusions

In this study, a novel hybrid ANFIS-MOPSO method for multi-objective optimization of machining parameters and GNPs reinforcement ratio was developed for milling GNPs-Ti64 matrix nanocomposites. First, GNPs-Ti64 matrix nanocomposites consist of 0 wt.%, 0.6 wt.% and 1.2 wt.% GNPs were successfully fabricated by the HFIH technique. Secondly, ANOVA-based response surface methodology was employed to examine the effects of the cutting speed, feed rate, and GNPs reinforcement ratio on the output responses including the depth force, feed force, and surface roughness. Third, artificial intelligence models based on ANFIS were developed and used as fitness functions for MOPSO. Finally, multi-objective optimization was performed using the ANFIS-MOPSO approach. The following main conclusions can be drawn.

- Optical microscopy images of the nanocomposites with 0.6 wt.%, and 1.2 wt.% GNPs show that GNPs are distributed uniformly and embedded in the Ti6Al4V matrix suppressing the grain growth during sintering. Moreover, the SEM backscattered images show the formation of the TiC particles between the GNPs and Ti6Al4V matrix particles.
- According to ANOVA results based on second-order quadratic models for each output response (F_d_, F_f_, and SR), it was found that cutting speed, feed rate, and GNPs reinforcement ratio are the significant factors.
- ANFIS is implemented successfully to predict and monitor the cutting force components (F_d_, F_f_) and surface roughness (SR) of the milled GNPs-Ti64 nanocomposites. The results showed that ANFIS model can obtain an accurate estimation of the depth force, feed force, and surface roughness with MAPE 3.87%, 8.56, and 2.21%, respectively. ANFIS models performed better estimation for all output responses compared with mathematical models.
- The ANFIS-MOPSO approach was combined for multi-objective optimization of F_d_, F_f_, and SR for the milled GNPs-Ti64 nanocomposites. The best single set of the optimal combination of milling parameters and GNPs reinforcement ratio could be obtained as cutting speed of 62 m/min, feed rate of 139 mm/min, and GNPs reinforcement ratio of 1.145 wt.% generating a depth force of 65.7313 N, feed force of 91.976 N and surface roughness of 0.215 µm. The ANFIS-MOPSO approach shows superior prediction performance as compared to the desirability approach available in Minitab. The predicted optimal sets of the machining parameters and reinforcement ratio can be used to achieve a higher quality of the machined surface, and minimum cutting forces, which lead to reduce energy consumption and a clean environment. At the same time, the current approach satisfies the Industry 4.0 manufacturing requirements by providing accurate prediction models to monitor and optimize the cutting conditions.

The developed hybrid ANFIS-MOPSO method can be extended for monitoring the tool wear during the milling process. Furthermore, the current approach can also be implemented for complex non-conventional machining processes, including laser and ultrasonic machining.

## Figures and Tables

**Table 1:** Composition of Ti64 powder.

Elements	Al	V	C	Fe	O	Ti
Percentage (%)	6	4	0.03	0.1	0.15	Balanced

**Table 2:** Characteristics of GNPs.

Power	Thickness	Average Diameter	Surface Area	Density
GNPs	5–8 nm	less than 2 µm	750 m^2^/g	2.21 g/cm^3^

**Table 3:** Machining parameters and their selected values.

Input Parameters	Values			
Feed Rate, F (mm/min)	90	150	210	-
Cutting Speed, V (m/min)	25	50	75	-
Reinforcement Ratio, R (wt.%)	0	0.6	1.2	-
Depth of Cut, d (mm)	0.5	-	-	-
Radial depth, d_R_ (mm)	3.8	-	-	-

**Table 4:** Hardness of the fabricated specimens.

GNP Reinforcement Ratio	Hardness (HV 0.5)
0	356 ± 6.686
0.6	503 ± 11.854
1.2	411 ± 4.828

**Table 5:** Experimental design and corresponding results.

	V (m/min)	F (mm/min)	R (wt.%)	F_d_ (N)	F_f_ (N)	SR (µm)
1	75	210	0.6	123.122	97.818	0.337 ± 0.0076
2	75	90	0.6	86.668	65.506	0.164 ± 0.0046
3	75	150	0	88.553	74.622	0.258 ± 0.0152
4	75	150	1.2	54.123	72.147	0.231 ± 0.0096
5	75	210	1.2	73.124	71.142	0.281 ± 0.0014
6	75	210	0	93.1259	81.124	0.397 ± 0.0035
7	50	90	0	103.467	81.402	0.336 ± 0.0139
8	50	150	1.2	60.623	92.243	0.272 ± 0.0069
9	50	90	1.2	55.328	51.715	0.343 ± 0.0066
10	25	90	0.6	183.270	130.857	0.364 ± 0.0132
11	25	210	1.2	203.250	141.282	0.406 ± 0.0095
12	50	90	0.6	146.101	82.809	0.189 ± 0.0112
13	50	210	0.6	175.141	108.220	0.417 ± 0.0033
14	75	150	0.6	82.275	71.981	0.265 ± 0.0029
15	75	90	1.2	33.093	36.491	0.279 ± 0.0091
16	50	210	1.2	78.211	99.835	0.321 ± 0.0256
17	25	90	1.2	68.289	66.082	0.292 ± 0.0062
18	25	210	0	225.828	162.197	0.54 ± 0.0140
19	50	150	0.6	108.715	136.168	0.283 ± 0.0010
20	25	150	0.6	275.408	138.010	0.374 ± 0.0070
21	25	210	0.6	255.722	192.410	0.471 ± 0.0081
22	25	150	0	189.967	128.962	0.411 ± 0.0052
23	25	150	1.2	177.208	104.014	0.348 ± 0.0039
24	50	150	0	97.101	99.241	0.374 ± 0.003
25	25	90	0	95.369	95.593	0.375 ± 0.012
26	75	90	0	65.854	74.780	0.269 ± 0.0150
27	50	210	0	131.129	114.121	0.462 ± 0.0068

**Table 6:** Results of the ANOVA for all selected output responses.

Output	Source	Degree of Freedom	Sum of Squares	Mean Squares	*p*-Value
Depth force	Model	6	91,361	15,226.8	0.000
	V	1	47,913	47,912.9	0.000
	F	1	15,648	15,648.5	0.000
	R	1	2988	2988.4	0.059
	Square	2	20,244	10,121.8	0.000
	V × V	1	4433	4433.4	0.024
	R × R	1	15,810	15,810.2	0.000
	2-Way Interaction	1	4567	4567.4	0.022
	V × F	1	4567	4567.4	0.022
	Error	20	14,876	743.8	
	Total	26	106,237		
Feed force	Model	6	27,006	4501	0.000
	V	1	13,573.6	13,573.6	0.000
	F	1	8145.7	8145.7	0.000
	R	1	991.1	991.1	0.058
	Square	2	2886.1	1443	0.01
	F × f	1	525.4	525.4	0.158
	R × R	1	2360.6	2360.6	0.006
	2-Way Interaction	1	1409.4	1409.4	0.026
	V × F	1	1409.4	1409.4	0.026
	Error	20	4885.4	244.3	
Surface roughness	Total	26	31,891.4		
	Model	6	0.168119	0.02802	0.000
	V	1	0.067149	0.067149	0.000
	F	1	0.057834	0.057834	0.000
	R	1	0.023429	0.023429	0.000
	Square	2	0.010943	0.005471	0.033
	F × F	1	0.006911	0.006911	0.034
	R × R	1	0.004032	0.004032	0.098
	2-Way Interaction	1	0.008764	0.008764	0.019
	F × R	1	0.008764	0.008764	0.019
	Error	20	0.026809	0.00134	
	Total	26	0.194928		

**Table 7:** Initial parameters for designing ANFIS models.

Responses	F_d_	F_f_	SR
Training Method	Hybrid	Hybrid	Hybrid
Membership Function	Trimf	Trimf	Trimf
Number of Membership Function	3 3 3	3 3 3	3 3 3
Number of Epochs	50	50	50
Output Function	Linear	Linear	Linear

**Table 8:** Selected parameters for designing ANFIS models.

Responses	F_d_	F_f_	SR
Training Method	Hybrid	Hybrid	Hybrid
Membership Function	Trapmf	Dsigmf	Pimf
Number of Membership Function	2 2 2	2 3 2	2 3 2
Number of Epochs	200	100	100

**Table 9:** Comparison of the developed models.

Output Responses	ANFIS Model	Mathematical Model (RSM)
	MAPE	MAPE
F_d_	3.87	17.85
F_f_	8.56	11.56
SR	2.21	8.32

**Table 10:** Parameters of MOPSO and machining constrains.

Parameters	Values
Population Size	30
Number of Iterations	30
Inertia Weight (w)	0.5
Learning Rate	0.7
Personal Learning Coefficient (C1)	1
Global Learning Coefficient (C2)	2
Machining Constraints	25 ≤ V ≤ 75 m/min 90 ≤ F ≤ 210 mm/min 0 ≤ R ≤ 1.2 wt.%

**Table 11:** Optimal milling parameter and reinforcement ratio settings.

Solution	Cutting Speed (m/min)	Feed Rate (mm/min)	Reinforcement Ratio (wt.%)	F_d_ (N)	F_f_ (N)	SR (µm)
A	75	90	1.167	35.894	36.495	0.2789
B	62	139	1.145	65.7313	91.976	0.215
C	75	90	0.678	79.8356	64.157	0.1542
D	67	90	0.678	99.534	63.772	0.1493
E	75	90	0.6328	108.9526	74.363	0.1317

**Table 12:** Optimal milling parameters and GNPs reinforcement ratio obtained by desirability approach.

Cutting Speed (m/min)	Feed Rate (mm/min)	Reinforcement Ratio (w.t%)	F_d_ (N)	F_f_ (N)	SR (µm)	Desirability
75	90	1.2	34.238	41.17	0.2287	0.9281

**Table 13:** Comparison of validation experiments for multi-objective optimization.

Parameters	Cutting Speed (m/min)	Feedrate (mm/min)	Reinforcement Ratio (w.t%)	F_d_ (N)	F_f_ (N)	SR (µm)
ANFIS-MOPSO	67	90	0.678	99.534	63.772	0.1493
Experimental	67	90	~0.6	91.54	68.17	0.163
	-	-	MAPE	8.7%	6.4%	8.4%
Desirability Approach	75	90	1.2	34.80	41.17	0.2287
Experimental	75	90	1.2	29.093	36.491	0.279
	-	-	MAPE	19.6 %	12.8%	18%

## Abbreviations

ANOVA	Analysis of variance
Al-TiC	Aluminium-titanium carbide
ANFIS	An adaptive neuro-fuzzy inference system
F_d_	Depth force
Dsigmf	Difference of two fuzzy sigmoid membership functions
F	Feed rate
F_f_	Feed force
FIS	Fuzzy inference system
GNPs	Graphene nanoplatelets
HFIH	High-frequency induction heating system
MMCs	Metal matrix nanocomposites
MAPE	Mean absolute percentage error
MOPSO	Multi-Objective particle swarm optimization
MFs	Membership functions
Ra	Surface roughness
R	Reinforcement ratio
Trimf	Triangular membership functions
Ti64	Ti6Al4V
V	Cutting speed
wt.%	Weight percentage
